# From the Approach to the Concept: One Health in Latin America-Experiences and Perspectives in Brazil, Chile, and Colombia

**DOI:** 10.3389/fpubh.2021.687110

**Published:** 2021-09-14

**Authors:** Christina Pettan-Brewer, Andreza Francisco Martins, Daniel Paiva Barros de Abreu, Ana Pérola Drulla Brandão, David Soeiro Barbosa, Daniela P. Figueroa, Natalia Cediel, Laura H. Kahn, Daniel Friguglietti Brandespim, Juan Carlos Carrascal Velásquez, Adolorata Aparecida Bianco Carvalho, Angela Maria Magosso Takayanagui, Juliana Arena Galhardo, Luiz Flávio Arreguy Maia-Filho, Cláudia Turra Pimpão, Creuza Rachel Vicente, Alexander Welker Biondo

**Affiliations:** ^1^Department of Comparative Medicine, School of Medicine, University of Washington, Seattle, WA, United States; ^2^One Health Brasil, Brazil, Brazil; ^3^Applied Microbiology Laboratory, Medical Sciences Department, Federal University of Rio Grande Do Sul (UFRGS), Porto Alegre, Brazil; ^4^Laboratory of Molecular Biology, Department of Parasitology, Veterinary Institute, Federal Rural University of Rio de Janeiro (UFRRJ), Seropédica, Brazil; ^5^Department of Preventive Medicine, School of Medicine, University of São Paulo, Brazil Ministry of Health, Brasilia, and Portal Saúde Única, São Paulo, Brazil; ^6^Department of Parasitology, Institute of Biological Sciences, Federal University of Minas Gerais (UFMG), Belo Horizonte, Brazil; ^7^Ecophysiological Modeling laboratory, Liberal Arts Faculty, Adolfo Ibáñez University and Applied Research Center of Chile (CIACHI) of Science and Education Foundation, Santiago, Chile; ^8^School of Agricultural Sciences, De La Salle University, Bogota, Colombia; ^9^Princeton School of Public Health and International Affairs, Princeton University, New Jersey and One Health Initiative Pro-Bono, Princeton, NJ, United States; ^10^Department of Veterinary Medicine, Rural Federal University of Pernambuco, Recife, Brazil; ^11^One Health Colombia, Veterinary Medicine and Zootechnics Faculty, University of Cordoba, Montería, Colombia; ^12^Department of Pathology, Theriogenology and One Health, School of Agricultural and Veterinarian Sciences, São Paulo State University (Unesp), Jaboticabal, Brazil; ^13^Environmental Health Laboratory, Department of Maternal-Infant and Public Health, School of Nursing, University of São Paulo, Ribeirão Preto, Brazil; ^14^School of Veterinary Medicine, Federal University of Mato Grosso Do Sul (UFMS), Campo Grande, Brazil; ^15^Department of Economics, Rural Federal University of Pernambuco (UFRPE), Recife, Brazil; ^16^School of Life Science, Pontifícia Universidade Católica Do Paraná (PUCPR), Curitiba, Brazil; ^17^Department of Social Medicine, Federal University of Espírito Santo (UFES), Vitória, Brazil; ^18^Department of Veterinary Medicine, Federal University of Parana (UFPR), Curitiba, Brazil; ^19^Purdue University, East Lafayette, IN, United States

**Keywords:** one health, ecohealth, planetary health, latin america, indigenous population, saúde única, salud unica, une seule santé

## Abstract

Professionals throughout the world have been working to assess the interdisciplinary interaction and interdependence between health and wellbeing in a constantly changing environment. The One Health concept was developed to encourage sustainable collaborative partnerships and to promote optimal health for people, animals, plants, the environment, and the whole planet. The dissemination of scientific discoveries and policies, by working directly with diverse communities, has been one of the main goals for Global One Health. The One Health concept has also been referred or related to as “One Medicine, One Medicine-One Health, One World-One Health, EcoHealth,” and Planetary Health,” depending on each fundamental view and approach. In Latin America, despite the concept still being discussed among health professionals and educators, several One Health initiatives have been used daily for more than decades. One Health action has been applied especially in rural and underserved urban areas where low socioeconomic status, lack of health professionals, and scarcity of medical resources may require professionals to work together. Local communities from diverse social and economic statuses, including indigenous populations have been working with institutions and social organizations for many years, accomplishing results through grassroots movements. These “bottom-up” socio-community approaches have also been tools for the prevention and control of diseases, such practice has preceded the One Health concepts in Latin American countries. It is strongly believed that collaborative, multidisciplinary, political, and economic initiatives with prosocial focus may become investments toward obtaining significant results in the face of global, economic and health challenges; working for a healthier world with inclusivity, equity, and equality. In this study, it is briefly presented how the One Health approach has been initiated and developed in Latin America, highlighting the events and actions taken in Brazil, Chile, and Colombia.

## Introduction

The One Health concept is not a new idea. Although historically, there have been times when medical doctors and veterinarians have worked together (1), it may be much of a generalization to conceive that such collaboration was common in the past. Indeed, one reason why Rudolf Virchow aimed for “one medicine” (later defined as One Health) in the 19th century was actually the lack of doctors and veterinarians working together (2). However, the 20th century brought greater isolation and separation between these two fields of knowledge (1). Considering the current accelerated global development, collaborative efforts and sustainable partnerships in a specific area should contribute to consistent strengths to reach relevant results, with applications directly in the areas studied and into the communities. This process occurred in several fields of global and population health, but the main area in which this idea is highlighted is the scientific research on medical topics.

A scientific and multidisciplinary approach for the health and wellbeing of humans and animals in a balanced environment, which results in the promotion of Planetary Health, showing that everything has been intrinsically connected (3). Also considering the growing interdependence between human beings and domestic or wild animals mainly due to food animal products and human-animal interactions, the medical and veterinary professions have been directed to work together within the collaboration scope toward wellbeing and global health (4). As a result, such an approach has encouraged studies to conduct sustainable partnerships between interrelated groups in different regions and continents to achieve optimal health for people, plants, animals, and the environment. This collaborative effort and holistic approach interactions for global One Health and environmental conservation have involved veterinarians, physicians, public health professionals, educators, anthropologists, environmentalists, and many other professions interconnected with communities. Although sometimes used as synonyms, the terms One Health, One Health approaches, EcoHealth, Planetary Health, One Welfare, and One Wellbeing represent different concepts linked to the same foundation. Some leaders in the field consider that the term, One Health, includes different approaches and differences among them. Since the topic is still controversial and open to discussion, further studies should establish a more stringent use of the said terms, which should be disseminated through teaching and training in all curricula worldwide. Regardless, a comparison of the three holistic approaches to health has been proposed (5), and One Health concepts may be given by practical examples, as already described (6).

## One Health History From Ancient Civilizations to the 21st Century in Latin America

### History of Health in Latin America Indigenous Population

The perception of health in humans and animals and knowledge of their interconnectedness can be long traced to the traditional knowledge of indigenous people in Latin America. Indeed, animals preceded humans in appearing in the territory by tens of millions of years, they have been deeply interconnected to the history of what is Latin America now. Human appearance has profoundly affected and shaped the health and life of native American animals, which subsequently led to a long history of the increasing human impact: from the Paleoindians, who may have caused the extinction of several Latin American megafauna species, to the Columbian Exchange that brought exotic species from the Old World, such as horses, cattle, sheep, dogs, domesticated American native species including turkeys, llamas, and alpacas, which brought extinction to several native American species. In such a similar dynamic scenario, animals have also influenced human history in an adaptive and interdependent human-animal relationship in Latin America (7).

Montenegro and Stephens (8) have thoroughly described indigenous health in Latin America. They clearly defined two periods of time: before and after the European invasion of the late 15th and early 16th centuries. These Latin American indigenous populations had complex cultures depending on the region they originated. The Inca, Aztec, and Mayan cultures had growing territories with urban populations, political, and military influences. More hunter and gatherer communities around the mountain and rainforests ecosystems were also observed, such as the Guarani in southern parts of South America.

Indigenous populations were neither static nor peaceful. Survival depended on war systems, different weapons, and food strategies. Health and wellbeing were intrinsically connected to sophisticated knowledge acquired through centuries regarding the balanced use of local ecosystems. European invasions changed the culture, inter-ethnic, and ecological relationships of natives. Health was also affected by new infectious diseases. For centuries, since the time of their colonization, conquest, or occupation, indigenous populations of tropical coastal environments suffered the most from illness and poverty. The Central Andes had a demographic collapse similar to the Bubonic plague epidemic in Europe in the 14th century. Later, such native populations have been affected by the continuous spread of diseases, habitat fragmentation, and land occupation associated with the lack of modern health care and infrastructure.

Although the use of animals in conventional medicine has been comparatively recent, a meta-analysis of historical and archaeological evidence indicated that animals have been used in traditional medicine in Latin America since ancient times. This was considered a “faunal drugstore.” Animals, mostly wild species, were used as both raw materials for clinically prescribed therapies, and as amulets and charms in native magic-religious rituals and ceremonies (9). Plants have also been used for both human and animal care in South and Latin America (10), demonstrating the environmental health impact on One Health. In contrast, animals have historically threatened human health prior to European arrivals, such as the Yanomami indigenous communities of Northern Brazil, which have been faced with high burdens of native soil, water and food borne zoonoses, including larvae of the native jigger flea, which causes severe disability of hands and feet (11).

Nowadays, many indigenous people still living within isolated environments have been constantly destroyed by non-sustainable agriculture and exploratory business, leading to harsh economic conditions, higher morbidity, and health risks. These populations have been connected and highly dependent on their local ecosystems for survival. Despite the accumulated knowledge and holistic understanding, One Health has much to learn about the early times of native Latin America, as the natural environment deeply influenced indigenous life, culture, and history.

### A Modern Historical Perspective of One Health in Latin American Countries

In 2010, the Food and Agriculture Organization (FAO), World Organization for Animal Health (OIE), and WHO collaboration officially established the One Health Tripartite (12). In addition, the European Union reaffirmed its commitment to operate under the One Health umbrella, and in 2011, the first International One Health Congress took place in Australia. In 2014, the International Society for Infectious Diseases (ISID) and ProMED, along with Skoll Global Threats Fund, HealthMap, and Training programs in Epidemiology and Public Health Intervention Network (TEPHINET), began working on another innovative tool for disease surveillance, namely, the EpiCore program. The EpiCore was created to build a network of field epidemiologists and health professionals who could validate reported and suspected disease outbreaks. ProMED moderators send requests for information (RFIs) directly to EpiCore members in a specific area of the world regardless of country or region. The specialties of the EpiCore membership reflect the One Health approach of ProMED with experts in animal, environmental and human health, all represented in the movement (13). Since then, increasing numbers of international organizations have promoted efforts to establish the One Health approach and actions around the world, including in Latin American countries (14–17).

Only in June 2021, at the 168th Session of the Executive Committee that the Pan American Health Organization (PAHO) has included One Health in the official agenda, as a “comprehensive approach for addressing health threats at the human-animal-environment interface” and prioritizing endemic diseases of zoonotic and vector-borne origin, emerging, and reemerging infectious diseases of zoonotic origin, antimicrobial resistance, and food safety (12). However, PAHO has promoted such a multisectoral approach, particularly in veterinary public health, for several decades, back to the Inter-American Ministerial Meeting on Health and Agriculture (RIMSA) in 1968 and 2016, the last entitled as “One Health and the Sustainable Development Goals”.

The PAHO proposed analysis and mapping of health interactions in specific national contexts, the establishment of One Health governance, strengthening multidisciplinary and intersectoral aspects, emergency preparedness and response, digital technology and scientific tolls, research, and capacity building as strategies in accordance with Agenda 4.6 One Health of the General Strategic Plan of PAHO for 2020-2025 with an estimated budget of the US $1 million per biennium (12). In that review, the PAHO has indicated a list of collaborating centers and best practices in One Health throughout the Americas, including the Collaborating Center on Environmental and Public Health at Fiocruz in Brazil, with best practices on leptospirosis and rabies approaches, and improvement surveillance on the triple-border area of Brazil-Argentina-Paraguay, and the Chilean Agency for safety and Food Quality (ACHIPIA) in Chile.

## One Health Experiences in Brazil, Chile, and Colombia

**Figure 1:**
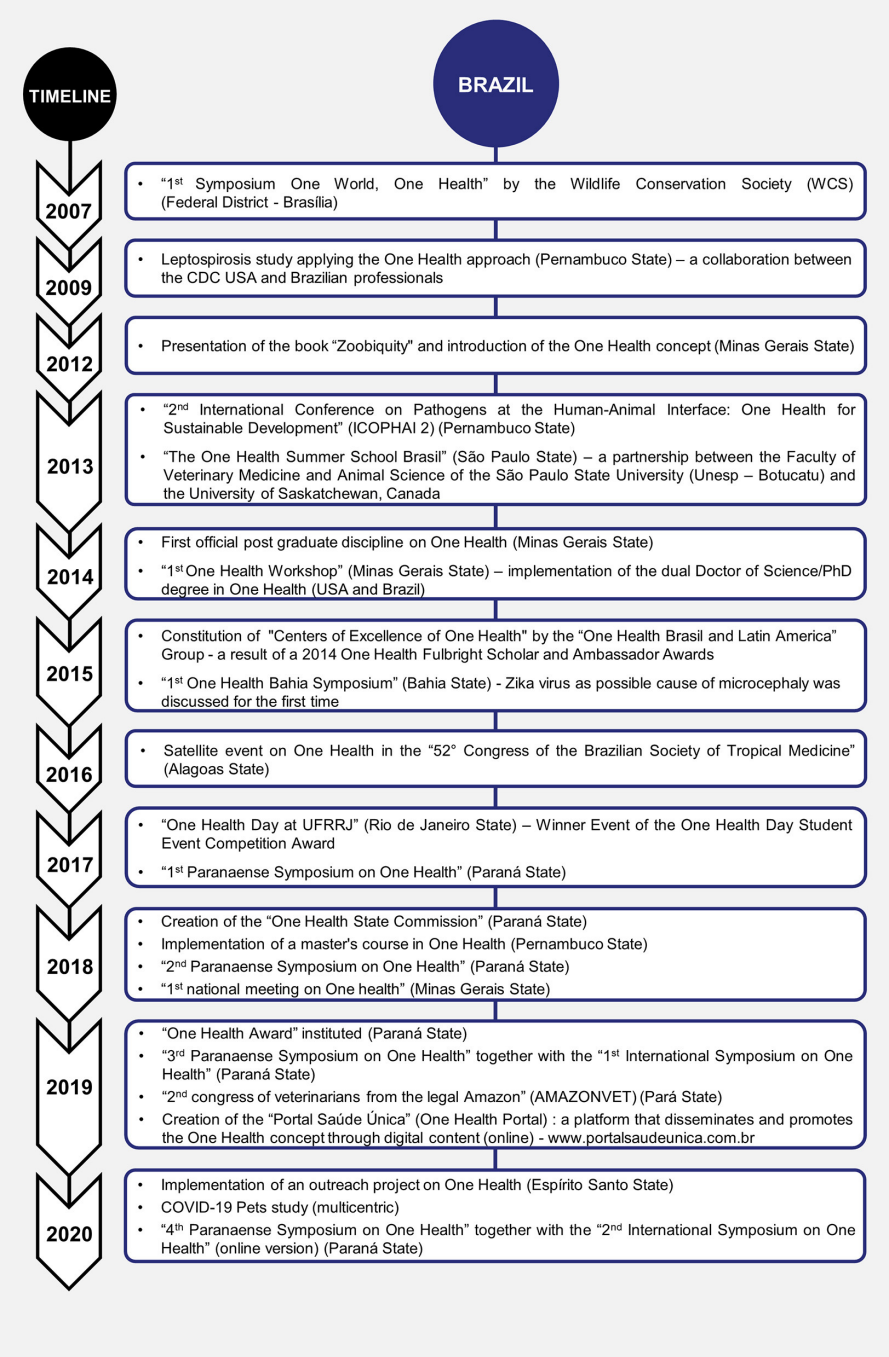
One Health history and development in the last decades in Brazil.

**Figure 2:**
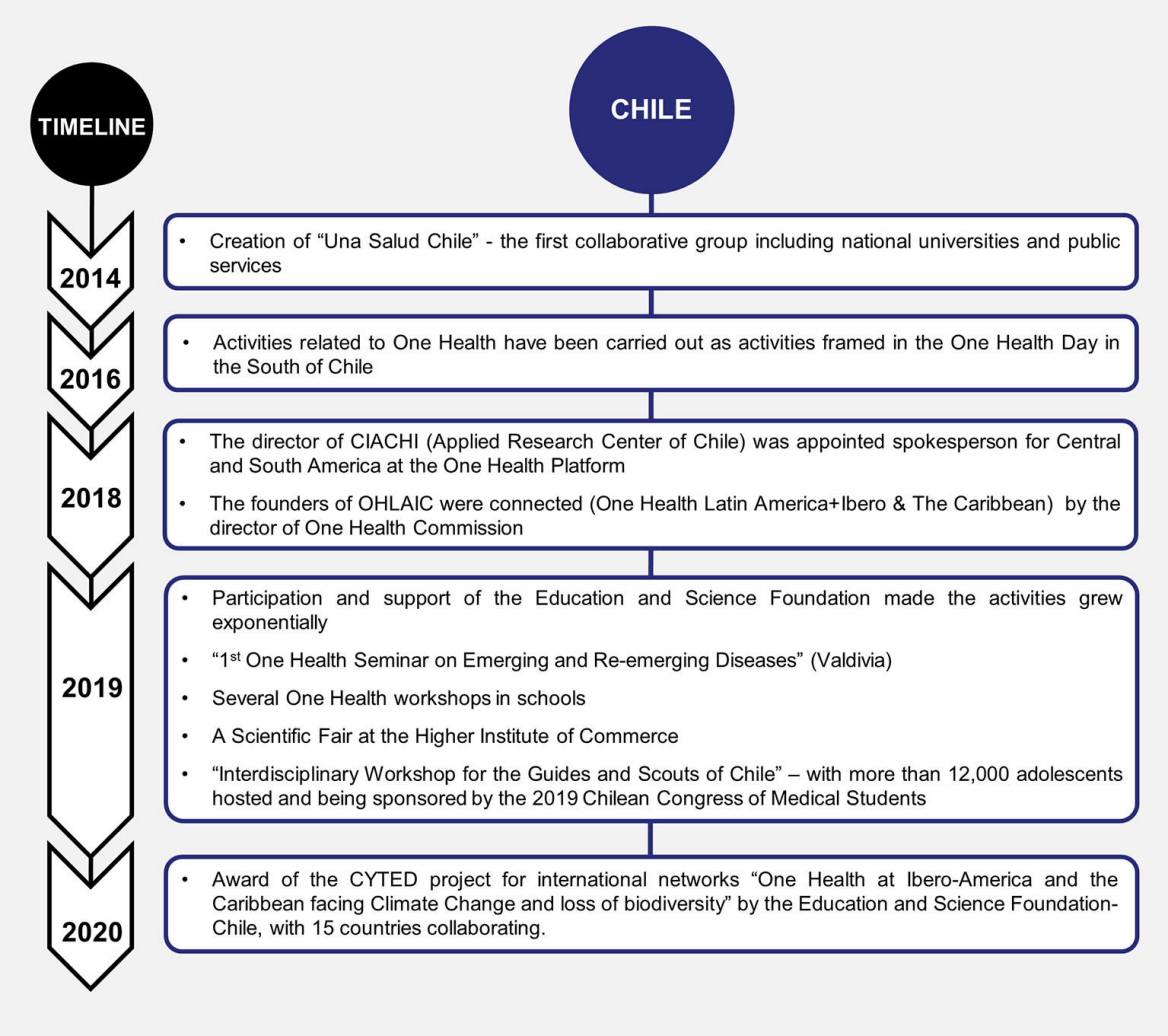
One Health history and development in the last decades in Chile.

**Figure 3:**
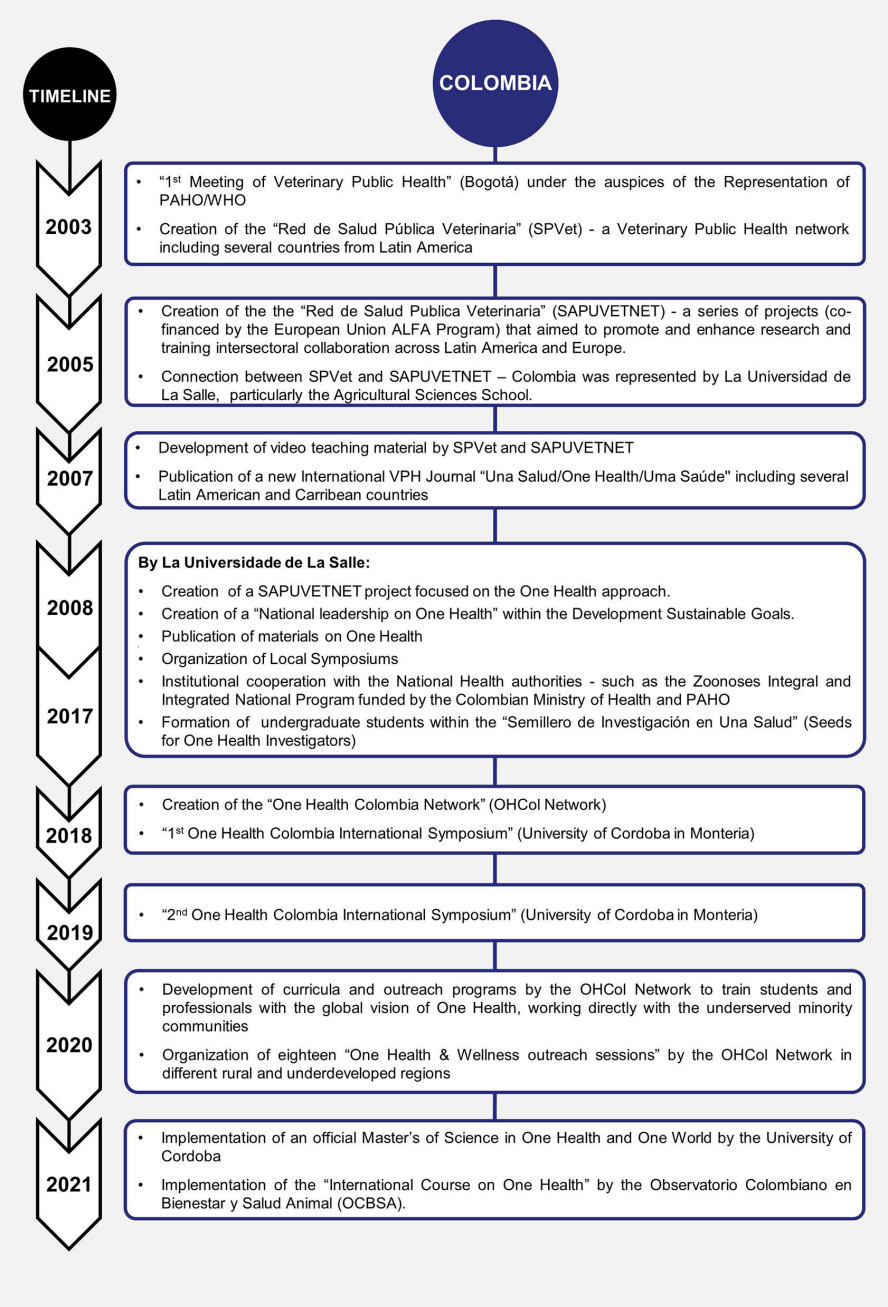
One Health history and development in the last decades in Colombia.

One Health history and development in the last decades in Brazil, Chile, and Colombia have been summarized and presented by timelines of each country (Figures 1–3, respectively).

### One Health in Brazil

#### From the Approach to the Concept – “Bottom-up” Grassroots Movements

One Health approach in Brazil has been reported long before the term One Health was coined. Since the beginning of Veterinary Medicine and Agriculture Schools in the 20th century, agriculture and health science professionals have been working together in indigenous, rural, and impoverished communities that had no access to health assistance. Conferences held by world organizations in Latin America supported the importance of interdisciplinary actions through Global Health. Wildlife preservation, habitat, and biodiversity topics had been the focus developed from national conservation institutions during the 20th and 21st centuries.

Since 2002, Veterinary Medicine students and residents, at São Paulo State University (Unesp) in Jaboticabal, northeast São Paulo state, Southeastern Brazil, have performed animal and public health outreach in rural communities which would later be regarded as One Health. Students accompanying community health agents assessed health risk factors related to the interaction between humans, animals, and the environment in homes and surrounding areas with attention to the main zoonoses as determinants of the health and disease processes in their ecosystems. Educational actions were applied, mainly, in primary and secondary schools with an expectation that children were the messengers for their parents and their behavior to be changed. All of these activities generate research for postgraduate studies.

The One Health movement in Brazil was officially introduced and recognized in 2007 when Dr. William B. Karesh, a wildlife veterinarian from the Bronx Zoo of New York, EcoHealth Alliance, and the Wildlife Conservation Society (WCS) led the first One Health Symposium in Brazil, introducing the theme “One World, One Health” in which wild animals act as important reservoirs and sentinels of diseases that affect human health being correlated with environmental destruction. In 2009, the Centers for Disease Control and Prevention (CDC) and Brazilian professionals collaborated with a Leptospirosis research study through the One Health approach in Northwestern Brazil. In December 2012, the concepts of One Health, EcoHealth, and Zoobiquity were introduced in Brazil by one of the authors of this study (CPB), who is also a zoo and wildlife veterinarian, by initiating and promoting One Health and spearheading official postgraduate research interdisciplinary programs among national and international academic institutions.

The Veterinary Public Health and Biotechnology (VPH Biotec) Global Consortium launched the “International Congress on Pathogens at the Human-Animal Interface (ICOPHAI).” The first edition occurred in Addis Ababa, Ethiopia, in 2011, and the second ICOPHAI was held in Porto de Galinhas, Brazil in 2013 to discuss issues related to zoonotic infectious diseases worldwide and thematic areas that necessitate One Health implementation in Latin America (16).

Since 2012, many One Health events have been officially established in several states and cities in Brazil. Most of the One Health events in Brazil were neither known nor advertised nationally or internationally. The Universities already had interdisciplinary outreach programs with One Health, such as approaches working directly with rural and diverse communities through the Unified Health System (“*Sistema Único de Saúde*” – SUS). By this time, the One Health concept was neither widely known nor advertised nationwide. Similarly, “The One Health Summer School Brazil”, which started in 2013, focuses on topics of infectious diseases, food safety, and public policies as part of an international collaboration between the School of Veterinary Medicine and Animal Sciences at Unesp, Prefeitura de Botucatu/SP, and the University of Saskatchewan, Canada. Postgraduate training was developed through international collaborations.

In 2014 and 2015, many actions were carried out in Brazil that contributed to the spread and application of the One Health approach in the country and Latin America. The first One Health Workshop was hosted in Minas Gerais State, Southeastern Brazil with national and international One Health experts (18). In the same year, The One Health Brazil Latin America Group was created and constituted by One Health Centers of Excellence (“*Centros de Excelência de Saúde Única*”) as a result of a 2014 One Health Fulbright Scholar and Ambassador Awards, spearheaded by one of the authors of this manuscript. The original Centers of Excellence at the time were held in the states of Bahia, Pernambuco, Minas Gerais, Rondônia, Pará, Roraima, and Mato Grosso do Sul. A successful example of these “grassroots” community and One Health approach movements through “*Centros de Excelência de Saúde Única*” occurred on March 5, 2015, with the First One Health Bahia Symposium held in Porto Seguro with attendance of more than 150 health professionals from different areas and institutions. Infant microcephaly that was possibly associated with the ZIKA virus was presented through the One Health concept by Brazilian physician infectologist, Dr. Antonio Bandeira, working together with Fundação Oswaldo Cruz (FIOCRUZ) and Hospitals. Similar activities involving directly community leaders were developed in these Centers of One Health.

Meanwhile, members of the One Health Brazil Latin America Association presented their work at the One Health Forum in Davos, Switzerland at the third Global Risk Forum One Health Summit 2015 (17), and at the first Global Conference on One Health (GCOH) in May 2015 in Madrid, Spain. This reinforced the commitment to continuing the dissemination of the concept and approach in Brazil and Latin America. The event in Madrid brought together researchers from 40 countries, with the participation of professionals from Brazil and Mexico. Partnerships survey of One Health Brazil Latin America were presented as a reference for several One Health projects in Brazil and Latin America.

The “One Health Brazil Latin America Association” became an official member of the World Veterinary Association (WVA) in 2015. The association included Brazil, Colombia, Peru, and Chile collaborators. Eventually, pioneer members from the Brazilian institutions reorganized and created the One Health Brasil with a purpose to unite, collaborate, organize, and centralize a sustainable multidisciplinary network of One Health, EcoHealth, and Planetary Health in Brazil.

After that, other important initiatives have been promoted, mainly by universities and professional associations like the 52nd Brazilian Society of Tropical Medicine Congress, held in Maceió, Northeastern Brazil in 2016. On this opportunity, the subject “Challenges for human and animal health in transforming ecosystems in a One Health perspective” was widely discussed.

The Centers of Excellence of One Health in Latin America were recognized internationally, supported by the World Veterinary Association (WVA), and the World Medical Association (WMA), receiving several Global One Health Awards in 2016–2017. The “*LeishNão* Project: A One Health approach for visceral leishmaniasis prevention in an endemic area in Brazil” by Galhardo et al. (19), “One Health in Brazil and the One Health International Project Programme” and “From the approach to the concept – a successful “grass root” One Health movement in Brazil and Latin America” by Pettan-Brewer et al. (17), received at the 33rd World Veterinary Association Congress in Seoul, Korea. In November 2016, the WVA, the WMA, the Japan Medical Association (JMA), and the Japan Veterinary Medical Association (JVMA) jointly held the Second WVA-WMA Global Conference (GCOH) on One Health in Japan following the inaugural GCOH held in Madrid, Spain in 2015. The proposal for the third WVA-WMA Global Conference (3rd GCOH) to be hosted in Brazil was presented by the One Health Brazil Latin America representatives and hosted by the One Health Brazil and the Veterinary and Medical Federal Associations. Unfortunately, the event was canceled due to the COVID-19 pandemic.

In February 2017, the antimicrobial resistance (AMR) group from the Pontifical Catholic University of Paraná (PUCPR), using the One Health approach, organized the round table, “Current state of Antimicrobial Resistance in Brazil and the United Kingdom,” together with the UK Science and Innovation Network and the School of Life Sciences of PUCPR. They invited 40 high-level experts from the government, academy, and private companies from the UK and Brazil to discuss the global and Brazilian state of AMR. As a result of this round table, several suggestions were made to improve the fight against AMR within the International Global Plan to fight AMR. In addition, the One Health Commission of the Regional Veterinary Council of Paraná State (CRMV-PR), a pioneer at the National level, was formed in April 2018 to fortify Veterinary Medicine in maintaining public, animal, and environmental health under the context of One Health. In 2019, through the State Commission for One Health, a partnership was established between the Federal and the Regional (PR) Council for Veterinary Medicine, and the first International Symposium on One Health was held, addressing zoonoses, disasters, mental health, and AMR. At the opening of the event, a term of commitment was signed between government entities, PUCPR, and the Regional Council to work in One Health. Thus, the School of Life Sciences (PUCPR), in 2019, changed all the matrices of its undergraduate courses, thus starting with One Health disciplines common to all courses.

After 2017, Federal and Professional Associations (CFMV, CRMV, COREM, CNS, CFM, SBM) have collaborated by disseminating the concept of One Health. The Preventive Medicine and Public Health sectors have always covered aspects of preventing and maintaining the health and wellbeing of animals and, by extension, of human beings. The conservation and preservation of the environment have shown great global interest in health and only in the last decades, with the new emerging diseases, and many enzootic epidemics have been proven to be associated with an imbalance in nature, destruction of habitats, and wild and domestic animals that were sentinels or reservoirs of new epidemics. An example of applying the One Health approach in endemic regions can be seen during the biggest epidemic of Yellow Fever in Brazil, which occurred in the years 2017 and 2018 due to a new cycle wave aggravated by mosquito-borne spreading, habitat encroachment, and exposure of the unvaccinated population. This occurred after almost 80 years of eradication in urban settings by vaccination in 1942. The fatality of wild primates during the epidemic demonstrated the importance of animals as sentinels for human health and the destruction of the environment associated with the reemergence of various zoonotic diseases (20).

Another aspect that highlights the spread of the One Health concept in Brazil and Latin America is the increasing number of events from these countries in the celebration of One Health Day (November 3) in the last years. This international campaign co-coordinated by the One Health Initiative, the One Health Platform Foundation, and the One Health Commission aims to bring awareness to the need for One Health interactions around the world. In 2018, an event organized by students from the Federal Rural University of Rio de Janeiro (UFRRJ) was one of the winners of the One Health Day Student Event Competition Award. The “One Health Day at UFRRJ” brought professionals from different health and environmental-related fields together to sensitize attendees from different backgrounds about the indissociable connection between human, animal, and environmental health. In the last years, similar educational events were held on a local, regional, and national level in Latin America, reinforcing the need for a multidisciplinary approach in many of the contemporary and future challenges.

From 2016 to 2021, other independent health groups continued in formation throughout Brazil and Latin America, such as courses and disciplines. The “1st National Meeting on One Health,” in Belo Horizonte, Minas Gerais State, Southeastern Brazil supported by the Federal University of Minas Gerais and Federal Council of Veterinary Medicine (CFMV) aimed to disseminate the concept “One Health,” its challenges, policies to professionals in the medical, veterinary, and environmental fields. Most of the One Health groups and events in Brazil initially concentrated on wildlife medicine, species preservation, environmental conservation (Planetary Health and EcoHealth), and emerging infectious diseases in Brazil and Latin America. Many groups focused on sustainable agriculture, agroindustry through the One Health approach, AMR, Food Safety, theater and arts, education, anthropology, and animal and human health, while others focused on comparative medicine and human-animal bond and wellbeing. Several professionals participate in these interdisciplinary partnerships, such as medical doctors, veterinarians, nurses, agronomists, nutritionists, psychologists, historians, anthropologists, statistics, biologists, dentists, conservationists, engineers, artists, and dancers.

Research groups in Brazil have also been applying One Health as a practical tool to solve problems such as zoonoses in different populations and their contact animals, taking advantage of the SUS, which allows comprehensive human-animal sampling. In such scenarios, wild boars, hunting dogs, and hunters have been surveyed in Brazil for vector-borne, waterborne, and foodborne diseases, for the first time worldwide (21). Moreover, One Health research and outreach community projects with community leaders toward Brazilian social classes vulnerability have provided interesting results in animal hoarders, homeless, incarcerated, indigenous, slum, low-income, and traditional island populations in their environments (22–25). In such a hands-on approach, companion and livestock animals have been concomitantly surveyed along with their owners, reaching holistic results, and establishing new roles in pathogen cycles of urban and anthropized settings.

#### One Health Training, Research, and Outreach in Brazilian Academic Institutions

The increase in the demand for qualification due to the requirements of the labor market has led health, agriculture and environmental professionals to increasingly seek a differential in their academic training with Higher Education Institutions. This fact was specially related to the insertion of different health professional categories, starting from the enactment of Law No. 8,080 of September 19, 1990 (26) in the area of Primary Health Care, more precisely in the Family Health Support Centers, following the publication of the Ministry of Health Ordinance No. 2,488, of October 21, 2011 (27).

Furthermore, the experience of interdisciplinary teaching, research, and extension of Environmental Health at the School of Nursing at Ribeirão Preto, University of São Paulo (SNRP-USP), began four decades ago in the training of clinical nurses. The history of this curriculum development had moments of coming and going, resulting from the institutional didactic and methodological options and trends. However, it took shape and acquired greater consistency over time despite the difficulties imposed by the traditional teaching model centered only on disciplinary practices and the hospital-centered care model. From the 1980s onward, there were important national movements with changes in health care proposals in Brazil, culminating in the promulgation of the new national constitution (1988) and strengthened by the Primary Health Care model and the SUS in 1990. The creation of an Interinstitutional Group for Studies and Research in Health Service Wastes (IGSRHSW) was strengthened by an interdisciplinary character, composed of professionals from different areas of knowledge and educational institutions, services and assistance in health, environment, sanitation, engineering, economics, and administration focused on Environmental and Planetary Health. Concomitant to the formation of this group, the Environmental Health Laboratory was created, in which an action project was established in the teaching, research, and extension to the community. Academic works were generated, including thesis, dissertations, scientific articles, manuals, books, and book chapters, among others, also offered to graduate students and postdoctoral researchers, opportunities for exchanges with relevant international institutions. Currently, the teaching of Environmental Health at SNRP-USP has been inserted in the teaching practice, in an interdisciplinary way and focused on One Health, through the proposal of building healthy and sustainable environments, aiming at training nurses and other professionals of the future for a globalized world.

In addition, since 2017, the class “One Health: Human, Animal, and Environment” has been offered to undergraduate students at the Federal University of Minas Gerais - UFMG, covering theoretical and practical aspects that include field visits in public parks and zoonosis control services in Belo Horizonte, Minas Gerais.

As previously mentioned, the introduction of the One Health concept in postgraduate education in Brazil was introduced, which was derived from the American term “One Health” and the interdisciplinary approach. The integrated and transdisciplinary training among professionals was brought to the fore among the professors of the Federal Rural University of Pernambuco (UFRPE), which was the utmost need to create and offer a outreach program that would meet the demands required by local communities and by the professionals working together in the Pernambuco State, Northeastern Brazil. Some professors realized in recent years, especially between the period of 2015 and 2018, that students entering postgraduate courses did not have the profile for training in graduate courses at the academic level because the vast majority of them already had employment in a public institution or private company linked to the health, agriculture or environment field, and were often interested in graduate programs mainly to update their knowledge and improve their performance in their professional fields or by their own need for changes in the field of activity in the companies in which they already operated.

In addition to this perception and concern from the professors, and due to the lack of a specific postgraduate program in One Health in the city of Recife, capital of Pernambuco State, the Professional Master's degree in One Health from UFRPE began to be designed and idealized by some professors, who took over the elaboration of the proposal, in the field of preventive veterinary medicine in the Department of Veterinary Medicine. However, since this department did not have enough professors who would meet all the demands of the minimum program content necessary for the training of students, other professors from other departments of the Federal Rural University of Pernambuco (UFRPE) and other institutions, namely, the Federal University of Pernambuco (UFPE) and the Federal University of Agreste of Pernambuco (UFAPE), were invited to collaborate. In addition, considering practical experience and experience in management and administrative positions, professionals from public institutions, such as the State Health Secretariat of Pernambuco, were also invited to compose the teaching staff and contribute to the training of professionals in the field of One Health.

Based on this demand raised by UFRPE, coordinating professors of the developing proposal have scheduled meetings with the various segments and institutions linked to the One Health field, such as municipal and state health departments and the Agricultural Defense Agency of the State of Pernambuco. The Defense Agency, in particular, readily showed interest and positively signaled support for the creation of a postgraduate course in the One Health field in addition to raising actions among their staff. This was done for training and updating, given the growing demands and needs from society for joint action of quality by the professionals who make up the staff of the respective companies. Thus, the implementation of a professional master's course in the field of? One Health at UFRPE was a demand needed not only by veterinary medicine professionals and other health professionals, given the different possibilities of performance in the professional market, but also by the society itself. This was done for technically qualified professionals to meet their needs, as food consumers and users of different health services, related to health surveillance, a better quality of life, animal health defense, environmental health, and primary health care.

Currently, the accredited professional master's degree in One Health at UFRPE has two lines of action and/or intervention called Surveillance and Primary Health Care and Epidemiology and Health Planning. Teaching staff formed by veterinarians, an economist, a dentist, a speech therapist, and, occasionally, professionals from other areas were invited to interact with the students, among which included social workers, nurses, physiotherapists, veterinarians, an administrator, a pharmacist, biologists, educators, among other professionals, who develop intervention projects in their institutions of origin, whether public or private, in the field of health, agriculture, and environment, related to health education, control of communicable or non-communicable diseases, water, food, management, processing of epidemiological data, AMR, among others at the intersection human, animal, and environment.

The design of shared disciplines at the post-graduate level by applying the Collaborative Online International Learning concept has also been an initiative to promote One Health among different professionals in Brazil and other countries (28). The Federal University of Espírito Santo (UFES), in partnership with Federal University of Paraná (UFPR), and universities from Mozambique (Catholic University of Mozambique), Germany (Ludwig Maximilian University of Munich - LMU - and Technical University of Munich), and Kosovo (Kolegji AAB), developed the “Joint Initiative for Teaching and Learning on Global Health Challenges and One Health” (JITOHealth) in 2020, financed by the Center for International Health at LMU. The JITOHealth targets education and training, focusing on surpassing the lack of collaborative approach, absence of cross-cultural experiences, and unequal distribution of scholarly resources in One Health, with experts from 22 institutions of the Americas, Africa, Europe, and Asia collaborating with the course content.

Outreach projects in higher education institutions also support the promotion of One Health among faculty, scholars, and communities in Brazil. An interdisciplinary team from UFES, recognizing the importance of training professionals in the One Health approach, created an outreach project entitled One Health ES in 2020, which involves faculty, professionals, and undergraduate and graduate students of different areas, such as medicine, veterinary, nutrition, pharmacy, biology, biomedicine, and dentistry. Periodically, the group meets to debate publications involving One Health and to plan and develop projects to be implemented in the community aiming at the health promotion and the prevention and control of diseases by applying the One Health concept, such as publication of informative material in social media. One Health ES also organizes webinars with invited experts, improving the network for further projects. The team also conducts research using this approach working directly with communities, which has been promoted in the social media of One Health ES. The engagement in the interprofessional actions, with collaborative participation of the entire team, highlights the promising impact of this initiative in the public health system and biodiversity in Brazil.

In Minas Gerais, Southeastern Brazil, the Post-Graduate Research Program (PPG) of the Federal University of Viçosa has been a program of international research partnerships with the University of Washington and The Paul Allen Global Animal Health at the Washington State University, the USA with an official dual Doctor of Science and PhD. The UFV/UW/WSU One Health interdisciplinary post-graduate partnership program continues to support sustainable collaborative research projects in One Health approaches as well as hosting exchange of professionals, students and visiting scholars.

During the COVID-19 pandemic, veterinarians in the Residency Program at UNESP Jaboticabal have been working directly in Public Health on different fronts of action both in Epidemiological Surveillance and Primary Health Care, as well as in the Coronavirus Service Center, performing telemonitoring of suspected patients. In parallel, two research projects have been developed: “Seroprevalence of SARS-CoV-2 infection in the municipality of Jaboticabal - SP: serial serological surveys” and “Molecular diagnosis of patients infected with SARS-CoV-2 in the municipality of Jaboticabal - SP: use of a safe and low-cost protocol.” Also, due to a departmental reorganization at the School of Agricultural and Veterinary Sciences, a Department of Pathology, Theriogenology and One Health has been renamed, demonstrating that One Health should be accepted as a reality that involves all disciplines related to animal health and global health, in the interface with the environment, including One Health in the curriculum, outreach, and research.

### One Health Brasil (OHB) Network History

As regional One Health groups were created, mainly through articulations in favor of research opportunities, but also for specific events, university professors and post-graduate students from different professional programs established a small online community in 2016 using the mobile platform, WhatsApp that would have become the most popular in Brazil and several countries. They established this community, the One Health Brasil Network, together with leaders and members of the One Health Brazil Latin America Association and several other One Health commissions and groups from all over Brazil. Initially, such a network aimed at simply sharing contacts and general information, intuitively associated with the themes of One Health, EcoHealth, and Planetary Health, from newspaper and scientific academic articles, policy reports, manuals, and regulations to advertise lectures and world events related to One Health.

Growing “organically,” especially from the outreach promoted by its members at symposia and conferences, the community began to face one of the great challenges of a community-based organization: effective communication. Often, the dialogical space was taken by manifestations that, although relevant, clearly diverged from the One health approach and, often, conveyed ideological positions and political readings of governmental programs and actions. With the recurrence of circumstances impairing constructive and collaborative dialogue, the necessity of a group management committee became clear. This management committee then started monitoring the posts and, whenever necessary, intervening to preserve the focus on One Health and, as much as possible, the predominance of collaborative attitudes.

In 2019, the management committee decided to create a website to what was already called the Rede One Health Brasil network (29), as a project. The creation of the website, itself, became an opportunity for the committee to enunciate the fundamental identity aspects of the organization, namely, its mission, vision, values, and strategic objectives. Next, the distribution of attributions and responsibilities in the first cycle of strategic planning was postponed with the emergence of the COVID-19 pandemic in the first quarter of 2020. On the one hand, One Health gained greater visibility from the global crisis. On the other hand, the process of consolidating presential partnerships and collaborations into new institutions seems to have been suspended, awaiting resumption in 2021. However, online meetings, lectures, discussion panels, and live webinars were happening daily. One Health Brasil network has been a successful example to all other countries of inclusive and sustainable interdisciplinary partnerships that unite a country through national and international collaborations. The network has established mutual official partnerships with organizations such as One Health Platform, One Health Initiative, One Health Commission, and One Health Sweden, continuing to build solid partnerships among uncountable international organizations from all continents. One Health Brasil also has thematic groups, such as ECOHA (*Ecossistemas Aquáticos: Saúde animal, humana e Ambiental*), an interdisciplinary subdivision applying One Health, EcoHealth, Planetary Health, and wellbeing of all lives in aquatic ecosystems.

In 2020, through the One Health Brasil network, investigators from different regions of Brasil received a CNPq Research Award (PetCOVID-19 Study applying the One Health approach) and have been leading the first SARS-CoV-2 research in pets in Latin America. Results have been notified to OIE-WOAH, peer-reviewed articles have been published, and education through media and internet tools have been emphasizing the importance of veterinarians as essential professionals in human health, animal welfare, and the prevention and control of pandemics through One Health.

### One Health in Chile

In 2014, the first initiatives of One Health began in Chile, forming the first collaborative group (*Una Salud Chile)* including national universities and public services (30). Subsequently, since 2016, some activities related to One Health have been carried out as activities framed in the One Health Day on November 3, which were reproduced in the South of Chile.

Later in Santiago, the education team of the Center for Applied Research of Chile (CIACHI) wanted to provide education in civil society and directly in the communities, working and consolidating the resilience of the villages within Santiago. On December 4, 2018, the director of CIACHI was appointed spokesperson for Central and South America at the One Health Platform and the director of One Health Commission connected the founders of OHLAIC. The activities grew exponentially throughout the year in Chile with the participation and support of the Education and Science Foundation. The First One Health Seminar on Emerging and Re-emerging Diseases was held in Valdivia, several One Health workshops in schools, and a Scientific Fair at Higher Institute of Commerce were carried out (31). Furthermore, an interdisciplinary workshop for the Guides and Scouts of Chile with more than 12,000 adolescents was hosted and sponsored by the 2019 Chilean Congress of Medical Students (32). In 2020, the first course in the world for non-biologist undergraduate students on One Health (law, psychology, journalism, and engineering) was given at the Adolfo Ibáñez University with excellent comments from students (33).

The main purpose of the National One Health Network (ReNOH) has been to connect all One Health groups in Chile to work based on local, national, and global objectives, including researchers and university students working in the same areas from all regions of the country. It is currently incorporating high school students and civil society, bringing the concept to schools and civic associations such as neighborhood councils or fairs.

The expansion of ReNOH has been a work in progress and discovery of new activities, in this context. Their main objectives have been to educate in One Health concepts and strategies, reaching the most remote and vulnerable communities (34). The main challenges for the groups that currently work in One Health in Chile have been to coordinate public policies under this concept and generate a greater national closeness and understanding of the scope of this strategy for university students of various careers and society in general, where until now there has been low penetration of the concept.

### One Health in Colombia

The history of the One Health approach in Colombia has also been linked to Veterinary Public Health teachings from the beginning of the 21st century. Professionals from several cities of the country gathered under the “*Red Salud Pública Veterinaria*” (SPVet). The SPVet network was created following a recommendation made at the First Meeting of Veterinary Public Health held in Bogotá (Colombia) in 2003 under the auspices of the Representation of PAHO-WHO. Organizations and Universities participating in this meeting included the National University of Colombia, Antioquia University, and the District Secretary of Health. During this meeting, an important dialogue related to food safety, prevention of zoonoses, poor public perception of the role of veterinarians in the wellbeing of the community, low importance of veterinary public health in higher education, and absence of guidelines for professional practices and the consequent fragmentation of the agricultural sector in the decision-making regarding the health system and the social and economic development of the country was held. The objectives of the SPVet network were the following: (1) maintaining a continuous and timely flow of information on veterinary public health topics, (2) strengthening ties of cooperation and support among specialists, create a space for discussion and consultation on topics of national interest as international, and (3) contributing to the strengthening of undergraduate and graduate academic activities in veterinary public health (35).

The need to integrate the improvement of professional activity and education in public health added to the need for veterinarians to take part in certain situations of emergencies affecting the relationship between humans and animals. This led to the development of the “*Red de Salud Publica Veterinaria*” (SAPUVET), a series of projects co-financed under the EU ALFA program, aimed to support an international network constituted by Faculties of Veterinary Medicine from Latin America and Europe. The projects have envisaged a series of objectives and activities aimed to promote and enhance research and training and intersectoral collaboration across Latin America and Europe. Project partners use a mail-list and distance learning platforms (e.g., Moodle, Colibri) to organize educational activities. Major results so far achieved have included the harmonization or development of a common curriculum, the creation of common modules on selected VPH topics, and that new teaching methodologies were used for a common training program on VPH, delivered *via* the Internet, using the problem-solving approach based on case studies. Challenges were experienced as a result of poor and unreliable internet connections. The use of modern communication and teaching methods in combination with written and theoretical material enabled lecturers and students at the universities involved to test, in some cases, for the first time, a problem-solving approach and a modular teaching structure in virtual format of real situations. The adoption of this innovative, flexible, less teacher-dependent mode of learning has played a key role in the activities of the network (36). Production of videos (DVDs) and self-learning software (CD-ROM) on meat inspection and hygiene (in three languages), development of an online VPH teaching Manual (beta version in Spanish), organization of e-conferences on upcoming VPH issues, publication of a new International VPH Journal “*Una Salud/One Health/Uma Saúde*,” (in three languages) exchanges of professors and researchers and coordinating meetings, participation in and organization of seminars, congresses, and conferences at the National and International level, and the publication of scientific and popular articles were used. The SAPUVETNET didactic tools have been tested and used by partner faculties and universities and other institutions. Didactic material can be freely circulated and distributed, used for distance learning, and be adapted to the local context of any country or geographical area, even outside Latin America and Europe (35–38).

The University representing Colombia in the SAPUVETNET project was La Universidad de La Salle, particularly, the Agricultural Sciences School, located in Bogotá. The school has been active for the past 10 years in (i.) publishing academic material, (ii.) promoting the One Health concept through participation and organization of local symposiums, (iii.) institutional cooperation with the National Health authorities such as the Zoonoses Integral and Integrated National Program funded by the Colombian Ministry of Health and Pan American Health Organization, and (iv.) formation of undergraduate students within the *Semillero de Investigación en Una Salud* (Seeds for One Health Investigators) (39, 40).

Another important network applying the One Health approach not only in the academic field but also in general communities is the University of Córdoba in Monteria, northern Colombia. This University constituted the One Health Colombia Network (OHCol Network) being recognized nationally and internationally by One Health Commission (USA) and the International Student One Health Alliance (ISOHA). This Institution held the first One Health Colombia International Symposium in 2018 and the second One Health Colombia International Symposium in 2019. The OHCol Network has been accredited as an official member of the interdisciplinary alliance for research and international collaborative training with the Schools of Medicine and Global Health, and the One Health Research Center (COHR) at the University of Washington, USA, and have been developing curricula and outreach programs to train students and professionals with the global vision of One Health, EcoHealth, and Planetary Health approaches for conservation and human, animal and environmental health among the underserved minority communities. Colombian health professionals working with research and community leaders through the One Health approach were also members of the original One Health Brazil Latin America Association which has been officially a member of the World Veterinary Association since 2015.

Since 2018, the One Health Colombia Network has held 18 “One Health and Wellness outreach sessions” in different rural and underdeveloped regions. More than 1,200 animals of several species have been evaluated, vaccinated, vitaminized, and dewormed. In addition, more than 7,000 people of all ages have received medical care and educational teachings in prevention health and welfare. These outreach sessions follow a human-animal-environmental interface methodological strategy based on Human Health activities, Biodiversity, and Economics Health (41). From 2020 to 2021, OHCol has developed One Health and One Welfare programs with institutions with other Latin American countries and also with One Health Centers from Colorado, Alaska, and Italy. An official Master's of Science in One Health and One World has been accredited at the University of Cordoba.

Under the COVID-19 pandemic, several Colombian public health research groups have published about the importance of the One Health Approach in the emergence of newer zoonotic infections like SARS-CoV-2 (42, 43). Authors have pointed out the potential of several zoonotic infections that calls for the implementation of One Health as a framework to design and operationalize better public health programs. Some of these groups were (i) the Epidemiology and Public Health Research Group at the De La Salle University in Bogotá, (ii) the Biodiversity and Ecosystem Conservation Research Group (BIOECOS), at the Fundacion Universitaria Autonoma de las Américas in Pereira, (iii) the Public Health and Infection Research Group, Faculty of Health Sciences, at the Universidad Tecnológica de Pereira, (iv) The Fundación Universitaria Agraria de Colombia (*Uniagraria*) located in Bogotá, v. CES University, Medellín, and vi. the National University of Colombia, located in Medellín and Bogotá.

One of the most significant contributions from the Colombian National Health Institute was the launching of the One Health Zoonotic Disease Prioritization workshop (OHZDP) in Bogotá in August 2019, based on the methodology developed by the CDC USA and Colombia (44).

In November 2019, the second International One Health Symposium held by the One Health Colombia and leaders from One Health in Latin America countries (Chile, Costa Rica, Cuba, Uruguay, Brazil, and others) signed a One Health Latin America Manifest for mutual understanding committing to work collaboratively and synergically within the One Health Latinoamérica, Ibero y el Caribe network (OHLAIC) (45).

### One Health in Latin America Ibero and Caribbean Network (OHLAIC)

Despite the adaptation of an English-speaking concept of One Health to Spanish, Portuguese, or French of most Latin American countries, the majority of terms can be easily translated due to their link to basic words describing almost the same health issues. However, Latin languages accept the switch of substantive-adjective. For example that “One Health” (*Saúde Única, Salud Unica, Une Seule Santé*) and “Health One” (*Uma Saúde, Una Sola Salud, Une Santé*), may be interchangeable in such languages.

**Figure 4:**
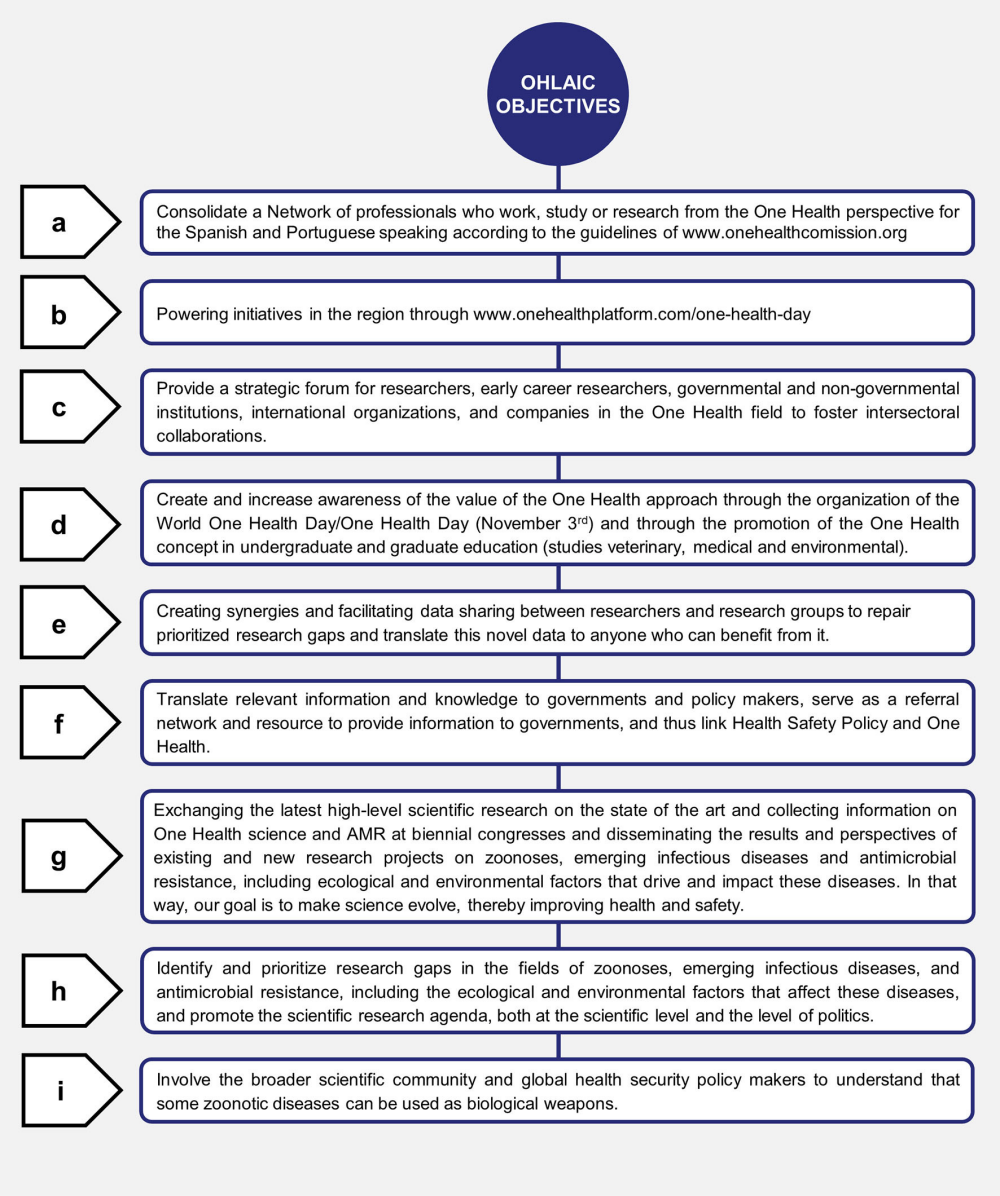
The objectives and goals of One Health Latin America, Iberia, and Caribbean Network.

One Health in Latin America arose after many researchers asked themselves why this concept was remarkably familiar and had not officially entered Latin America. The mutual experiences were that it was due to idiosyncrasies, language barriers, political and economic difficulties. For this reason, individual countries started building and strengthening One Health Networks with professionals who already were working with a One Health approach without distinction of race, creed, political ideology but inclusive networks at no cost and with the consideration that in Latin American countries the budgets for research and education were very low. Hence, each country in Latin America has different experiences and the history of each one remains to be fully described. The objectives and goals of the One Health Latin America, Iberic, and Caribbean countries network have been detailed and presented in Figure 4.

In 2019, a “*Quien es Quien*” *in One Health in Latin America* Webinar was presented and hosted by the One Health Commission with over 17 countries representing Latin America and the Caribbean Islands (46). In December 2019, the Network OHLA (sounding as Hello! in Spanish or Portuguese) grew with the addition of Spain and Portugal, changing its original name to OHLAIC to include Ibero and Caribbean countries (47). In April 2020, the OHLAIC webinar cycle began with the first Webinar “Reflections on the COVID-19 pandemic from the vision of One Health and One Welfare” (48) continuing in July 2020 with the second webinar “COVID-19: SARS-CoV2 disease and wildlife in Latin America” with excellent exhibitors and a large audience. Thus, consolidating a network with clear objectives and that in December 2020 a CYTED project (Ibero-American program of science and technology for development) was awarded for thematic networks on the subject of sustainable development and climate change for 4 years (49). Recently, during December 8–11, 2020, SAPUVETNET-OHIN and Latin American Universities held a webinar series organized by the Universidad Peruana Cayetano Heredia in Lima presenting multiple results of research studies within the One Health Approach.

#### Next Steps for One Health in Latin America and the Caribbean With the World

The One Health in Latin America, Ibero, and Caribbean Network has presented specific objectives which have been gathered, summarized, and presented in Figure 4. However, the specific goals of One Health networks in Latin America rely on each country and their prioritized actions. In such One Health approach, main goals have been accomplished by collaborative synergism and non-competitiveness, sensitizing different society actors, culture inclusiveness and diversity, and practical multidisciplinary working groups. The objectives of these One Health networks in Latin America have prioritized actions from a collaborative synergism and non-competitiveness to work from a One Health approach to sensitize the different actors of society and to form working international groups in the different areas of global health. It has served as a platform for meeting and bringing together different professionals linked to different areas of human, animal, and ecosystem health, and for allowing the exchange of not only scientific knowledge, but also the union of different cultures, thoughts, and initiatives around One Health. There has been a great importance to interculturality in such a way as to know the roots of all Latin Americans and try to preserve them and keep them active over time, even more so now to generate a new deal with the ecosystems as the native peoples did with respect, equality, passion, and wisdom.

Another main objective of such networking is the establishment of a standard system for the joint assessment of international infectious disease risks, construction of sustainable mechanisms for collaboration and communication between the bodies and ministries responsible for human health and animal health, and align national, regional, and international strategies. This is to prevent and control diseases with collaboration and participation in an intersectoral initiative implementing the concept in daily practice. One Health offers a systematic approach to complex problems that involve interactions between spheres of human-animal-environmental health. This approach has been increasingly important in an era of rapid changes in the environment, including climate change. It requires new types of transdisciplinary collaboration, direct participation with local communities to conduct integrated assessments, and interventions that consider the interconnected health of humans, animals, and the environment. As described in the introduction, One Health terms represent different concepts linked to the same One Health foundation. However, it has been essential to continue the discussion for the welfare of human beings and animals, the connection between all (One Welfare; One Wellbeing) (50) and the complex integration of One Health, Health of the Environment (EcoHealth), Economics Health, and Health of the Planet (Planetary Health) as a remarkable continuing global agreement by all countries following its 17 Sustainable Development Goals and 169 targets for the future health of the planet and all life on it (51, 52).

## Discussion

To further develop the One Health concept, we must consider taking into account the accelerated advances in science and the globalization of our economies. This has been important because about 75% of emerging infectious diseases have been shared between humans and domestic and/or wild animals. Emerging zoonotic diseases that affect Latin American countries today result from interactions between natural and human-animal-plant systems. Infectious agents, such as *Salmonella* spp., *Escherichia coli*, tuberculosis, malaria, yellow fever, influenza A(H1N1), West Nile Virus, Zika, Dengue, Chikungunya, and SARS-CoV-2, have been examples in which animals, humans, and the environment have been intrinsically related. In these cases, animals were also victims of the emerging infectious diseases transmitted by vectors being sentinels for human health and a sign of an imbalance in the environment, especially by habitat destruction due to deforestation or pollution. In addition, emerging or neglected zoonotic diseases, such as hantavirus pulmonary syndrome, leptospirosis, trypanosomiasis (Chagas disease caused by *Trypanosoma cruzi* and other species), brucellosis, hanseniasis, treponemas, and leishmaniasis arose when human beings have invaded forest region, increasing the contact between people wild animals who might act as pathogen reservoirs. A better understanding of the type of contact between human and animal populations (domestic or wild) has been fundamental for modeling how zoonotic infections will emerge and spread. The emergence of several unidentified species of new pathogens in Latin America and co-infection with *Bartonella, Ehrlichia*, and *Rickettsia* as possible etiologic agents of anthropozoonoses and zooanthroponoses, and related diseases in pets, livestock, and wildlife have intensified the interest in neglected and emerging pathogens.

Zika, dengue, hanseniasis, leptospirosis, leishmaniasis, yellow fever, parasitic and other neglected diseases in Brazil, and the complex nature of emerging zoonotic diseases demonstrated the need to strengthen even more interdisciplinary training and partnerships elucidating the concept of One Health in Latin America and support the work with indigenous tribes and underprivileged communities. As a result of collaborative efforts supported by Latin America's One Health, networks have developed and organized national teams for disasters preparedness through One Health. On February 11, 2021, Latin American representatives from the WHO contacted the One Health Brasil network executive members to collaborate in the submission of a proposal for the importance of One Health in Pandemics, Food Safety, Food Security, and Bioterrorism.

The emergence and spread of diseases with sustained transmission from person to person have long hit humanity, representing challenges for science and public health, worldwide. Changing natural environments can modify the balance between species, increase contact between them and establish human-animal bridges. Other activities, such as wildlife trafficking, incorrect use of soil or water, urbanization without sustainability, habitat destruction, lack of basic sanitation, fires and deforestation, and the absence and/or breaking of health protocols can also contribute to the emergence of new diseases. Although governments have been responsible for public health policies, the multifactorial character in the emergence of pandemics means that the responsibility for controlling those that have been occurring, and for preventing new ones from arising, be that of all professionals, or future professionals, and of citizens living in a community. It has been the role of collaborative efforts to promote discussions and generate and disseminate knowledge, fulfilling their role in the training of professional citizens, since only knowledge can lead to changes in habits and behaviors of individuals and society.

One of the challenges, foremost, in the academic scientific world is to understand the importance to work together equally with the native and minority populations of Latin America by listening to what these ancient cultures have to teach the scientists and not vice versa. Politics, years of lack of trust built among conflicting cultures, excessive bureaucracy and biased government policies from these countries interfere with collaborations and research partnerships to be developed. The One Health approach creates a unique effort to promote Unity in Diversity, cultural coexistence, human-animal-plant-ecosystem harmony, and spiritual universal values. Their Unity in Diversity concept that we share the same sky, walk the same Earth, breathe the same air, and that we are a single family might bring resolution for successful partnerships in a more holistic health vision. This unity is the essence of life from ancient civilizations and indigenous peoples which must be learned, as animals and the natural environment have deeply influenced indigenous life, culture, and history, in an adaptive and interdependent relationship throughout Latin America.

The recent experience of Latin America with COVID-19 has demonstrated the importance of a more interconnected world through cooperative and less competitive collaborations. One Health may be the way for preventing future pandemics and health disparities instead, focusing on health equity, environmental and economic sustainability. Not surprisingly, through One Health initiatives, veterinarians with all professions have been associated at the front response of COVID-19 pandemics. One Health may represent the main future strategy and basis for preventive actions requesting veterinarian leadership, as potential unknown animal pathogens may still become emergent in human beings (53, 54). Latin America has been an example of “hands-on” One Health actions for a more inclusive and healthier Planet from the approach to the concept with more equal representation.

There were some limitations to this review. Primarily, the review has relied mostly on personal knowledge of authors and search of main selected events instead of a formal “One Health” systematic review, a relatively new concept word in Latin America which would fail to provide a comprehensive search of both indexed and non-indexed studies, experiences, and events. Although the authors have focused the review on only three Latin American countries, such an international approach has not been published to date, and it may stimulate and inspire future interactions and publications by One Health researchers in neighboring countries worldwide. Thus, the review herein has aimed to establish a starting point by rescuing the history and current efforts on One Health initiatives in only three out of the 33 countries currently established in Latin America and the Caribbean to date.

### Importance of One Health

Although the past topic has offered a detailed discussion about One Health experiences and conferences held at different times, the importance of One Health should be described separately. The strengthening of national and international collaborative partnerships in elucidating the concept of One Health by sharing experiences in conferences and during on-field practical actions has led to a better implementation of the One Health approach and measures to mitigate, control and prevent emerging and infectious diseases, such as Zika, dengue, hanseniasis, leptospirosis, yellow fever, antibiotic-resistant bacteria and parasitism, and the complex nature of diseases in indigenous and underprivileged communities in many Latin American countries.

The One Health approach supports global health security by improving coordination, collaboration, and communication at the human-animal-plant-environment interface to address shared health threats, such as zoonotic diseases, antimicrobial resistance, food safety and security among others. Examples of potential benefits and outcomes of One Health actions are to monitor and develop standards at the local, national and global health equity, ensure adequate capacity in health including strategies to prevent, detect and respond to outbreaks of diseases, develop emergency preparedness responses, support interdisciplinary collaborative partnerships, and control of highly infected pathogens and reemerging diseases, and conduct scientific research.

One Health is an inclusive collaborative, multisectoral, and transdisciplinary approach, working at the local, regional, national, and global levels, with the goal of achieving optimal health outcomes recognizing the interconnection between people, animals, plants, and their shared environment. Furthermore, the Importance of a One Health approach has increased in advancing global health security and the Sustainable Development Goals.

Finally, improvement of international, national, regional and local levels of One Health networking and collaborative research studies, associated with professional training, conferences, and continuing education, has led to a better understanding of the human-animal interactions (domestic or wild) and spatial modeling on emerging, reemerging, and spreading of zoonotic infections. The advance of One Health in concepts and approaches in such a holistic way has provided key tolls for prevention and preparedness, as already presented at the discussion for tuberculosis, malaria, yellow fever, influenza A(H1N1), West Nile Virus, Zika, Dengue, Chikungunya, and recently for SARS-CoV-2.

## Conclusion

Independent of the approach used to describe the link and vision of Health of the Environment (EcoHealth), Economics Health, Health of the Planet (Planetary Health), and Population Health to One Health, all these concepts share the same goals as understanding their use as practical holistic tools for better and more effective solutions to address threats to the health, wellbeing and sustainability of humans, animals, plants and the environment. Above all, One Health can be used as a preventive measure for upcoming threats not only for Latin America but for the shared planet Earth as a whole.

Based on local “grassroots” demands, action movements and population needs, the experiences of One Health history and development in Latin America and Caribbean countries are different from other continents, from within the same continent like North America, and even among the three Latin American countries discussed herein. This review hopes to inspire other countries, regions, and continents to also share their own One Health experiences.

While the One Health approach worldwide is considered crucial to address governance challenges of complex issues and is widely supported in theory, its implementation in practice remains limited, especially due to lack of financial support and the secular anthropogenic and self-centered mentality. Altogether, the world must continue working to further establish a standard system of equal assessment of health, construction of sustainable mechanisms for collaboration and communication between responsible agencies and ministries for human, plant, and animal health. Furthermore, align national, regional, and international strategies with collaboration, cooperation, and intersectoral partnership implementing the concept of environmental conservation in daily practice. Not only to learn and change the paradigms of health and diseases, of economic-political-cultural crises for the conservation of the Planet's Biodiversity with inclusion, equity, and sustainability with a holistic view, but also change our Ego into the Eco framework. Finally, there is a critical need to respect, rescue, and learn from diverse communities and indigenous peoples who have the ancestral knowledge for a balanced life on our planet. Thus, future One Health experiences in Latin America should always associate cross-disciplinarity action and concept, surpassing political, social, cultural, and linguistic differences.

## Author Contributions

All authors contributed equally, approved the submitted version, and collaboratively to the written manuscript. CP-B designed the original manuscript. Figures were designed by DF, CV, NC, and AB. AM, DA, DSB, DFB, DF, AB, and CV finalized the review.

## Funding

The University of Washington Faculty grant 75-5324 PETTAA was applied for publication fees.

## Conflict of Interest

The authors declare that the research was conducted in the absence of any commercial or financial relationships that could be construed as a potential conflict of interest.

## Publisher's Note

All claims expressed in this article are solely those of the authors and do not necessarily represent those of their affiliated organizations, or those of the publisher, the editors and the reviewers. Any product that may be evaluated in this article, or claim that may be made by its manufacturer, is not guaranteed or endorsed by the publisher.
